# The Development of Systemic Inflammatory Diseases in Hidradenitis Suppurativa

**DOI:** 10.3390/diagnostics13030502

**Published:** 2023-01-30

**Authors:** Natsuko Saito-Sasaki, Yu Sawada

**Affiliations:** Department of Dermatology, University of Occupational and Environmental Health, Fukuoka 807-0804, Japan

**Keywords:** hidradenitis suppurative, systemic organ, inflammation

## Abstract

It is understood that the skin is a peripheral lymphoid tissue that defends against external environmental stimuli. Continuous activation from these factors, on the other hand, promotes persistent inflammation at the local location and, occasionally, tissue damage. Hidradenitis suppurativa (HS) is a typical inflammatory skin disease and becomes a source of numerous inflammatory cytokines due to the chronic intractable repeated inflamed tissues. Because inflammatory cells and cytokines circulate throughout the body from the inflamed organ, it has been hypothesized that HS-mediated skin inflammation impacts the systemic functioning of numerous organs. Recent updates to clinical and experimental investigations revealed that HS has a significant connection with systemic inflammatory disorders. We provide the details and comprehensive molecular mechanisms associated with systemic inflammatory illnesses due to HS.

## 1. Introduction

Peripheral lymphoid immune cells interact with other organs to protect against external environmental invaders or chemical exposures for a host’s defense [1,2,3]. Inflamed peripheral lymphoid organs become the source of activated immune cells and inflammatory cytokines, enhancing the host’s immune system and sharing information regarding the external environmental factors that should be recognized as danger signals [4,5]. Although these interplays are essential for the host’s defense, the excess inflammatory reaction sometimes exacerbates undesired tissue damage in the peripheral tissues [6,7], including the primary peripheral lesion in addition to distant organs.

Hidradenitis suppurativa (HS) is a chronic recurrent debilitating inflammatory skin follicle disease that severely impairs a patient’s quality of life [8,9]. HS is recognized as the background for the activation of innate immunity in the inside of hair follicles. After puberty, painful inflammatory lesions occur in the deep layers of the skin at the site where apocrine glands are found [10,11]. Indeed, HS skin lesions are commonly observed in the axilla’s, inguinal, anogenital, and buttocks’ hair follicles [12].

Recently updated investigations revealed that cutaneous chronic inflammation has the potential to enhance systemic organ dysfunction mediated by skin-derived inflammatory actions [13]. Indeed, representative inflammatory skin diseases, such as atopic dermatitis and psoriasis, showed various systemic organ dysfunctions, such as cardiovascular disease [14,15]. Because of the characteristics of HS as an intractable recurrent cutaneous chronic skin inflammation, it has been speculated that HS-derived inflammatory response drives systemic inflammatory reaction in various organs. In this review, we focused on the HS-mediated systemic organ influences based on the etiology of HS and HS-related inflammatory responses to figure out the whole HS-mediated pathological influence of these systemic organ dysfunctions.

## 2. Etiology of HS

**Figure 1:**
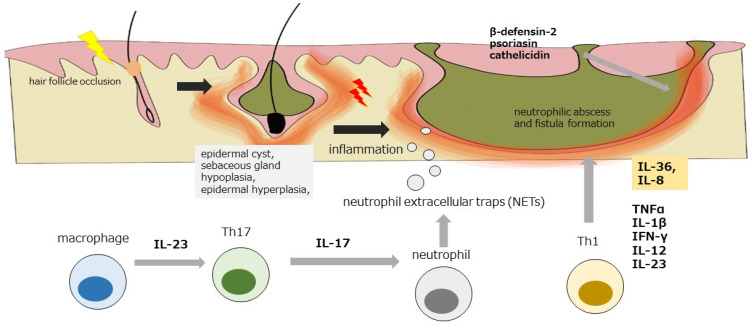
The pathogenesis of HS. The hair follicle occlusion is the initial step for the development of HS; subsequently epidermal cyst, sebaceous gland hypoplasia, epidermal hyperplasia, neutrophilic abscess, and fistula formation may develop, leading to the formation of a subcutaneous complex network in each fistula. TLR2 expression was enhanced in macrophages and dendritic cells, which abundantly express the inflammatory cytokines IL-12 and IL-23. IL-23 is thought to be involved in Th17 cell infiltration in lesions. Other various inflammatory cytokines such as TNF-α, IL-1β, IFN-γ, IL-12, and IL-36, are expressed in lesions. The production of antimicrobial peptides, such as β-defensin-2, psoriasin, and cathelicidin, is also enhanced in lesions.

Since HS frequently occurs in apocrine gland-dominant skin sites, it is speculated that apocrine glands have some influence on the onset [16]. Hair follicle occlusion is the initial step for the development of HS [17] (Figure 1). Subsequently, patients may develop an epidermal cyst, sebaceous gland hypoplasia, epidermal hyperplasia, or neutrophilic abscess and fistula formation, leading to the formation of a subcutaneous complex network in each fistula. In severe chronic cases, foreign body granuloma reaction and infiltration of B cells and plasma cells were also observed [18]. 

The infiltration of neutrophils, macrophages, and dendritic cells is seen in the initial inflammatory reaction in HS. Toll-like receptor 2 (TLR2) expression was enhanced in macrophages and dendritic cells, suggesting the possibility of microbial-derived substances serving as ligands for the induction of inflammation [18]. Macrophages abundantly express the inflammatory cytokines IL-12 and IL-23 [19]. IL-23 is involved in lesional Th17 cell infiltration [20], which induces further inflammation by releasing neutrophil extracellular traps (NETs) consisting of nuclear chromatin and granules from neutrophils [21]. Various inflammatory cytokines, such as TNF-α, IL-1β, IFN-γ, IL-12, IL-23, and IL-36, which are thought to be related to Th1 and Th17 cells, are expressed in lesions [22]. In addition, increased expression of IL-36, a member of the IL-1 family, is observed in lesions and is involved in the pathology of HS through the induction of IL-8 [23]. The production of antimicrobial peptides, such as β-defensin-2, psoriasin, and cathelicidin, is also enhanced in lesions [24]. These inflammatory cytokines directly induce various forms of tissue damage [25,26,27]. Interestingly, TNF inhibitors have been reported to reduce these inflammatory cytokines [28]. 

## 3. HS and Systemic Inflammatory Diseases

This section summarizes the association with HS and systemic inflammatory diseases such as cerebrocardiovascular diseases, renal dysfunction, liver dysfunction, Alzheimer’s disease, osteoporosis, chronic obstructive pulmonary disease (COPD), inflammatory eye disease, inflammatory bowel disease, hypothyroidism, alopecia areata, psoriasis, psychological depression, obesity, and cancers. In addition, the possible pathogenesis of HS-mediated inflammatory responses is summarized. 

### 3.1. Cerebrocardiovascular Diseases

Cardiovascular events in inflammatory skin diseases are significant events for clinicians, and it is important to understand to what degree HS-mediated inflammatory reaction is involved in the risk of cardiovascular diseases. The risk of cardiovascular diseases due to HS has been reported in various countries and areas, indicating that the influence of HS on cardiovascular diseases is crucial for patients with HS.

A population-based retrospective cohort analysis of 6779 patients with HS in Israel found that patients with HS had a higher risk of myocardial infarction (hazard ratio (HR) 1.33; 95% confidence interval (CI): 1.04–1.68) and a higher risk of dying from myocardial infarction (HR 12.56; 95% CI 7.59–20.80) [29].

A population-based cohort study of 5964 patients aged 18 years or older with HS in Denmark found that the highest incidence rate ratios (IRRs) were 1.57 for myocardial infarction (95% CI: 1.14–2.17), 1.33 for ischemic stroke (95% CI: 1.01–1.76), 1.95 for cerebrovascular disease-associated death (95% CI: 1.42–2.67), and 1.53 for major adverse cardiovascular events (95% CI: 1.27–1.86) (MACE) [30]. 

A larger population of 49,862 individuals with HS in Denmark was also the subject of a retrospective cohort investigation [31]. Patients with HS showed an increased risk of myocardial infarction (HR, 1.21; 95% CI, 1.12–1.32) and cerebrovascular events (HR, 1.22; 95% CI, 1.14–1.31) [31]. Younger patients had the highest relative difference in composite myocardial infarction or coronary artery disease risk compared to controls (18–29 years: 1.67; 95% CI, 1.37–2.03) [31].

Asian populations also showed a similar influence of HS in cerebrovascular diseases. Population-based cohort research of 478 newly diagnosed HS patients in Taiwan found a greater risk of coronary artery disease (HR: 2.722, 95% CI: 1.628–4.553) [32].

Carotid intima-media thickness is an indicator of the future risk of myocardial infarction and was assessed by carotid ultrasonography in 68 individuals with HS in Spain [33]. Carotid plaques were considerably more common in HS patients compared to control (HS, 30.9% vs. control, 22.1%) [33]. HS was shown to be substantially associated with the occurrence of carotid plaques in a multivariable regression model (OR: 2.99, 95% CI: 1.26–7.13) [33].

A prospective observational study using carotid ultrasonography evaluated subclinical atherosclerosis in 62 HS patients in Spain [34]. Subclinical atherosclerosis was seen in 30.6% of HS patients and 16.1% of controls [34]. HS patients aged 40 and above had a higher risk of subclinical atherosclerosis (OR: 4.9, 95% CI: 1.8–13.1) [34]. These changes showed the direct influence of the presence of HS on the development of atherosclerosis.

As the mechanism of HS-mediated cerebrovascular or cardiovascular diseases, IL-1 cytokines are triggered by various environmental factors, and NLR family pyrin domain containing 3 (NLRP3) is highly expressed in atherosclerotic plaques. Il-1β is released from cholesterol crystals in the plaques [35]. The IL-1β blockade of canakinumab impairs inflammation in blood vessels in patients with cardiovascular disease [36] and reduced recurrent cardiovascular events [37].

In individuals with coronary atherosclerosis, IL-17-producing cells were found in peripheral blood and atherosclerosis in the coronary artery [38]. Overexpression of epidermal IL-17A exacerbated endothelial dysfunction [39]. These cytokine-induced endothelium damages enhanced TNF-α- and IL-17A-induced cerebrocardiovascular disease development.

### 3.2. Renal Dysfunction

Various studies showed evidence of an association between many inflammatory skin diseases and chronic kidney diseases. Psoriasis and atopic dermatitis have associations with reduced kidney function [40]. A case-control analysis of 56,602 individuals with chronic kidney disease (CKD) in stages 3–5 in the United Kingdom found that they were more likely than controls to have a history of HS (odds ratio (OR): 1.49, 99% CI: 1.19–1.85) [40]. Because renal dysfunction severity tends to depend on the severity of other inflammatory skin diseases [40], it has been speculated that patients with severe HS are more likely to develop severe renal dysfunction.

Although the molecular basis of HS-related renal impairment is unknown, it has been postulated that an inflammatory process in the skin drives chronic inflammation in the kidney. Th17 frequency was higher in patients with end-stage renal disease [41], and IL-17A-expressing cells depended on the abundance of inflammatory cytokine production and were associated with the degree of renal injury. On the contrary, IL-17A inhibition impaired renal dysfunction [42]. Similarly, mice lacking IL-17A were protected from renal damage. According to these investigations, IL-17 leads to the development of renal impairment [43]. IL-17 is produced in the skin in patients with HS, suggesting that IL-17-mediated inflammatory action is one of the pathogeneses of renal dysfunction in HS.

### 3.3. Liver Dysfunction

Representative liver dysfunction in the current situation is a nonalcoholic fatty liver disease (NAFLD) which encompasses two separate disorders with unique histologic characteristics and prognoses: nonalcoholic fatty liver (NAFL) and nonalcoholic steatohepatitis (NASH). Furthermore, NASH is a very severe inflammatory disorder that can lead to fibrosis and end-stage liver disease (ESLD). Several inflammatory skin diseases, including psoriasis, have been linked to an increased prevalence of nonalcoholic fatty liver disease (NAFLD) [44]. Recently, updated studies showed that the presence of HS is associated with the risk of these liver dysfunctions.

A retrospective analysis of 51 patients with HS alone, 20 patients with HS plus NAFL, and 12 patients with HS plus NASH in the United States showed that NASH and NAFLD were found in a significant percentage of HS patients [45]. The average HIS4 score among HS plus NASH patients was the highest (average: 12.7), whereas it was comparable among HS alone and HS plus NAFL patients (average: 9.6 and 9.4, respectively) [45], suggesting that the severity of HS is closely associated with the frequency of co-existence of NAFLD.

A European country also showed the same characteristic of HS in association with NAFLD. A case-control study of 70 HS patients in Spain found that the prevalence of NAFLD was substantially higher in HS patients than in controls (72.9% vs. 24.7%) [46]. According to a multivariable regression model, HS was substantially and independently linked with the occurrence of NAFLD (OR = 7.75; 95% CI: 2.54–23.64) [46].

Another study including 125 patients with HS from Spain revealed that these individuals had a considerably greater prevalence of NAFLD than non-hidradenitis suppurativa patients (57.6% vs. 31.7%) [47]. An independent link between hidradenitis suppurativa and NAFLD was established by multivariable analysis (OR: 2.79, 95% CI: 1.48–5.25) [47].

As the mechanisms, Th17 cells may play an important role in NAFLD pathogenesis, according to emerging findings. High-fat-fed mice as an NAFLD animal model had a greater IL-17-producing cell frequency in the liver [48,49]. An IL-17 neutralization reduced liver damage [50]. IL-17A-inhibiting antibodies prevent NASH and, later, HCC [51]. NASH development was prevented in mice lacking IL-17 [52]. These findings indicate that HS-derived skin inflammation enhances the development of NAFLD in an IL-17-dependent manner.

### 3.4. Alzheimer’s Disease

Several studies indicated that γ-secretase complex mutations have been linked to familial HS and Alzheimer’s disease (AD). In the brain, γ-secretase cleaves the amyloid precursor protein, leading to the creation of the β-amyloid plaques that cause Alzheimer’s disease [53]. In mouse skin, genetic inactivation of γ-secretase generates epidermal and follicular histologic abnormalities similar to those reported in HS, which are thought to be driven by Notch signaling alterations [54]. In addition, γ-secretase has been identified as the genetic foundation of sickness in a subset of familial HS patients [55]. 

Research including 192 HS patients in Turkey showed that the prevalence of Alzheimer’s disease was observed to be considerably greater in HS patients with a family history of HS (25.8%) than in HS patients without a family history of HS (6.2%) [56]. The most remarkable finding of this study is a 4.5 times greater risk of AD in HS patients with a family history of HS, which rises to 8.8 times in individuals over 40 years old (CI: 1.54–13.37) [56].

Another study of 28,755 individuals with HS in the United States showed that HS was related to a higher risk of Alzheimer’s disease (OR: 1.23, 95% CI: 0.96–1.56) [57], suggesting that HS itself is a risk factor for the development of Alzheimer’s disease.

In addition to γ-secretase-mediated pathogenesis, HS-mediated immunological reaction has been speculated to be the trigger for Alzheimer’s disease. The number of Th17 cells was shown to be considerably elevated in Alzheimer’s disease patients [58]. Alzheimer’s disease mice models injected with Alzheimer’sAmyloid1-42 (A1-42) demonstrated an infiltration of Th17 cell in the brain [59]. Furthermore, elevated interleukin (IL)-17 and IL-22 levels were seen in the hippocampus [59]. The Fas ligand was shown to be associated with neuronal apoptosis and was preferentially expressed by Th17 cells [59]. Furthermore, therapy with anti-IL-17 antibodies reduced A1-42-injected neurotoxicity and pro-inflammatory cytokine production while improving memory performance [60]. Adalimumab impaired this inflammatory response via lower NF-B activity, which was significant in neuroinflammatory transcription factors [61]. These findings suggest that HS-mediated immune condition plays a part in the pathogenesis of Alzheimer’s disease.

### 3.5. Osteoporosis

Inflammatory skin diseases enhance bone fracture or reduced bone mineral density due to those related to inflammatory cytokines [13,62]. HS has also been recognized as a risk factor for future events of bone fracture. A cross-sectional study of 32 HS patients in Denmark showed HS patients had lower muscular and bone mass percentages [63]. Another cohort study including 39 HS patients in France population showed spondyloarthritis was detected in 28.2% of patients with HS, indicating a high risk of HS (OR: 11.0, 95% CI: 4.1–83.3). The most prevalent spondyloarthritis, seen in 81.8% of individuals with HS, was axial spondyloarthritis [64]. A total of 81 HS patients in Spain enrolled in a study that showed that HS patients had a lower trabecular bone score and total hip bone mineral density compared to controls [65]. These findings indicate the association of HS-mediated pathogenesis of osteoporosis.

As the mechanism of HS-mediated bone fragility, TNF-α has a role in the development of osteoporosis in HS. TNF-α increased bone resorption by activating osteoclasts [66]. In addition, IL-6 increased bone resorption [66]. Furthermore, IL-17 contributes to osteoporosis. IL-17 is essential for the formation of osteoclasts. Anti-IL-17 treatment decreased osteoclast development considerably [67]. Furthermore, IL-23 promotes bone loss in T cells via enhanced osteoclastogenesis and receptor activator of kappa B ligand (RANKL) expression [68]. IL-23 stimulated tartrate-resistant acid phosphatase (TRAP) in osteoclasts, which accelerated osteoporosis [68]. Indeed, combining anti-IL-23 with anti-IL-17 antibodies reduced osteoporosis and stopped bone loss [68]. Although no studies have been carried out on the subject, systemic medication for HS may be effective in preventing osteoporosis due to HS.

### 3.6. Chronic Obstructive Pulmonary Disease (COPD)

COPD is closely associated with the pre-existence of inflammatory skin diseases. Recent studies showed that HS also becomes a risk factor for the future occurrence of COPD. A cohort analysis of 5306 HS patients in the United States found that the most common comorbid disorders among matched HS patients were chronic pulmonary illnesses (1540 cases (40.3% of frequency)) [69]. A total of 448 individuals who self-reported having an HS diagnosis in the Netherlands showed that COPD was substantially linked with HS (OR: 1.74, 95% CI: 1.35–2.23) [70].

COPD development is aided by Th17 and Tc17 [71]. Pattern recognition receptor expression, including that of TLR3 and TLR7, was increased by IL-17 [72], resulting in a highly sensitive inflammatory response to pathogens, suggesting that HS-derived IL-17 might also be associated with future risk of COPD.

### 3.7. Inflammatory Eye Disease

A study involving 20 participants in the United States showed that 65% of patients had uveitis, 30% had scleritis, and 5% of patients had peripheral ulcerative keratitis [73]. A single-center retrospective study of 236 patients with HS in the United States revealed 22 cases (9.3%) had inflammatory eye diseases. The most frequent kind of inflammatory eye disease was anterior uveitis (40.9% of the individuals with inflammatory eye diseases) [74]. However, the detailed risk of HS for inflammatory eye disease remains unclear. IL-17 and TNF-α were increased in uveitis lesions [75]. IL-6 and IL-23-deficiency reduced inflammation of autoimmune uveitis [76]. A vitamin D analog of calcitriol reduced uveitis development as well as the generation of IL-17 [77].

### 3.8. Inflammatory Bowel Disease

A probable association between HS and inflammatory bowel diseases has been demonstrated in several studies. A study including 7732 individuals with HS in Denmark found that the prevalence of Crohn’s disease in HS patients and the general population was 0.8% and 0.3% (OR: 2.04, 95% CI: 1.59–2.62), respectively, and the prevalence of ulcerative colitis in HS patients and the general population was 1.3% and 0.7% (OR: 1.75, 95% CI: 1.44–2.13), respectively [78]. Patients with HS had a considerably higher frequency of developing new-onset Crohn’s disease (HR: 2.19, 95% CI: 1.44–3.34) and ulcerative colitis (HR: 1.63, 95% CI: 1.18–2.27) [78].

IL-17 is strongly linked to intestinal inflammation. Transferring T cells with IL-17A or IL-17F deficiency into T cell-deficient animals resulted in colitis [79]. IL-21 is required for Th17 differentiation in the gut. Th17 differentiation could not be induced in IL-21-deficient animals or those with IL-21-neutralizing antibodies [80]. These data imply that Th17 positively drives gut inflammation, leading to the development of Crohn’s disease [81].

### 3.9. Hypothyroidism

Previous research has found a link between HS and thyroid disease. A higher risk of hypothyroidism was found in HS patients (OR: 2.91, 95% CI: 2.48–3.40) in a study conducted in Israel including 4191 HS patients [82]. 

Because the mechanisms of IL-17 relate to the risk of hypothyroidism in HS patients, circulating peripheral Th17 cell frequency was dramatically elevated in patients with Hashimoto’s disease [83]. FT4 was negatively linked with the levels of IL-17 and IL-23 [84]. TNF-α might also be important for the development of hypothyroidism. A case study demonstrated the case of a juvenile idiopathic arthritis patient who, during adolescence, also had autoimmune Hashimoto’s thyroiditis. The patient was given etanercept, which improved thyroid function [85]. These findings show that HS-driven inflammatory cytokines may amplify inflammatory reactions in the thyroid, which then produces a risk of hypothyroidism.

### 3.10. Alopecia Areata

Alopecia areata is a prevalent type of immune-mediated hair loss in which the hair follicle is attacked by an autoimmune process, resulting in non-scarring hair loss. Several studies have found an increased risk of alopecia areata in HS patients.

A Korean study of 28,516 HS patients found that alopecia areata was more prevalent in HS patients than in non-HS patients (OR: 1.35) [86].

A cross-sectional analysis of 3645 HS patients in the US population also found that the HS group had a substantially greater risk of AA (RR = 2.09; 95% CI, 1.69–3.20) [87].

Inflammatory cell infiltrates around the bulbar region of hair follicles in alopecia areata, the cause of hair loss [88]. Aside from Th1, Th2 cytokines, particularly IL-13, are elevated in alopecia areata [89]. Alopecia areata causes an increase in IL-23 and CD4+. IL-17+ cells infiltrate surrounding hair follicles in the acute phase [90]. These data suggest that activation of Th2, in addition to Th17 cells, play a role in the development of alopecia areata, possibly due to HS-mediated inflammatory reactions. 

### 3.11. Psoriasis

Psoriasis a representative inflammatory skin disease; the common key cytokine IL-17 is involved in the pathogenesis of psoriasis and HS. A systematic review analysis of 3 cohort studies, 1 case-control study, and 6 cross-sectional studies found that patients with HS had a 2.67-fold increased risk of psoriasis (95% CI, 1.84, 3.87). Male patients with HS had a 4.30-fold greater incidence of psoriasis than male patients without HS (95% CI, 2.37, 7.78) [91]. Since IL-17 is a major player in the pathogenesis of psoriasis, the background of Th17-dominant immunological situations in HS might become subject of future study regarding psoriasis. 

### 3.12. Depression

Inflammatory skin diseases are closely associated with the future risk of psychological depression. Not limited to the manifestation of inflammatory skin diseases, recent updated study showed the possible influence of cutaneous inflammatory cytokine association for the development of psychological depression. 

A Danish population of 7732 patients with HS revealed that HS patients had an increased risk of completed suicide (HR: 2.42, 95% CI: 1.07–5.45) [92].

Retrospective cohort analysis of 49,280 adult and 3042 pediatric patients with HS in the United States showed that adults and pediatric patients with HS had a 10% (HR, 1.10; 95% CI, 1.07–1.13) and 26% (HR: 1.26, 95% CI: 1.10–1.44) increased risk of developing depression compared to control individuals, respectively [93].

Chronic stress increased the ratio of activated pro-inflammatory T helper 17 (Th17) in the liver and ileum in a mouse model of chronic, unpredictable, moderate stress-induced depression [94]. This animal model also demonstrated elevated IL-17, and anti-IL-17 treatment reduced anxiety and depression-like behavior [95]. Furthermore, Serum IL-4 and IL-13 levels were also greater in a group with a serious depressive illness [96]. Treatment with dupilumab reduces anxiety and depression symptoms [97,98]. These findings suggest that HS-derived inflammatory cytokines may raise the risk of mental illnesses and that systemic medication may be beneficial in preventing the development of psychological disorders.

### 3.13. Obesity 

Metabolic syndrome has been identified as a comorbidity in HS patients. Studies focused, in particular, on the increasing prevalence of obesity. Significantly, a meta-analysis revealed that the OR of obesity among HS patients was 3.5 (95% CI, 2.2–5.4) times higher than that of controls [99]. 

The direct impact of HS on obesity is unknown. However, along with the mechanism of delayed clearance of inflammatory reactions, obesity itself worsened IL-17-mediated skin inflammation when a high-fat diet was consumed [100,101,102], suggesting that obesity might contribute to the development of HS.

### 3.14. Skin Cancer

HS influences the development of various malignancies. The prevalence of primary liver cancer (standardized incidence ratio (SIR), 10.0; 95% CI, 2.1–29.2) and nonmelanoma skin cancer (SIR, 4.6; 95% CI, 1.5–10.7) was higher in a group of 2119 HS patients in Sweden [103]. In a Korean population of 22,468 individuals with HS, the HR of total cancer was 1.28 (95% CI, 1.2–1.4). Patients with HS showed a substantially greater risk of Hodgkin lymphoma (HR, 5.1; 95% CI, 1.2–21.4), oral cavity and pharyngeal cancer (HR, 3.10; 95% CI, 1.60–6.02), central nervous system cancer (HR, 2.4; 95% CI, 1.2–4.7), nonmelanoma skin cancer (HR, 2.1; 95% CI, 1.1–3.8), prostate cancer (HR, 2.1; 95% CI, 1.3–3.2), and colorectal cancer (HR, 1.5; 95% CI, 1.1–1.9) [104]. 

Chronic inflammatory responses shift the immune environment toward Th2-dominant settings. Th2 immunity is essential for oncogenesis and is implicated in the suppression of antitumor immune responses [105], implying that the ongoing skin inflammatory response in HS may potentially serve as a trigger for the occurrence of systemic organ malignancies.

## 4. The Summary of the HS-Related Inflammatory Reaction for the Development of Systemic Inflammatory Diseases

**Figure 2:**
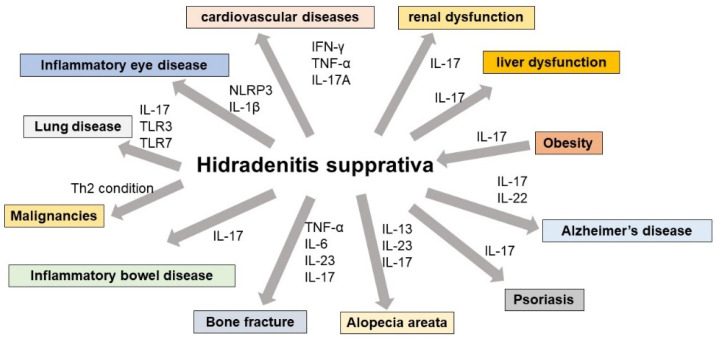
The influence of HS-mediated inflammatory response in systemic organ dysfunction. HS-mediated inflammation contributes to the development of systemic organ dysfunctions. HS lesional skin drives various inflammatory cytokines which exacerbate inflammatory responses in other organs. This figure shows the HS-related risk of systemic organ dysfunction and associated inflammatory cytokine for the development of these pathogeneses.

We summarized the interaction of HS-related inflammatory reactions for the development of systemic inflammatory diseases. Figure 2 depicts the overall interplay of HS-mediated inflammation with various organs. Because the human body’s skin interacts with other organs, the features of HS as a severe cutaneous inflammatory response impacts the excess inflammatory response in systemic organ involvement. Systemic medication, particularly biologics, can consistently limit the systemic organ dysfunction mediated by HS-triggered inflammatory responses, indicating the relevance of managing the cutaneous inflammatory response in HS patients. Thus, the common inflammatory reactions between HS and systemic inflammatory diseases may become a highlighted issue to determine the importance of systemic therapy.

## 5. Conclusions

This review outlines the information gained from recent research attempting to understand the actual risk and possible pathogenesis of HS-related inflammatory reactions for the future risk of systemic inflammatory organ diseases. On the other hand, there are a limited number of studies that have elucidated the risk of other HS-mediated inflammatory diseases, and further investigation of the risk of other various inflammatory diseases in patients with HS is desired. In addition, other inflammatory skin diseases have revealed that other types of organ-derived inflammation constitute a risk for future inflammatory skin disease. Therefore, the entire interaction of peripheral lymphoid organ-mediated inflammatory reaction also needs to be clarified.

As one of the important issues in this topic, it remains unclear whether HS-targeted therapies influence HS-mediated systemic organ inflammation. In inflammatory skin diseases, psoriasis and atopic dermatitis influence the development of other systemic inflammatory diseases and their treatment diminishes the future risk of other inflammatory organ diseases [60,106,107], suggesting that HS-targeted treatment might reduce the risk of other HS-related inflammatory organ diseases. The therapeutic impact on other systemic organ inflammation diseases must also be examined in further investigations.

## Data Availability

Not applicable.

## References

[1] Campione E., Lanna C., Diluvio L., Cannizzaro M.V., Grelli S., Galluzzo M., Talamonti M., Annicchiarico-Petruzzelli M., Mancini M., Melino G. (2020). Skin immunity and its dysregulation in atopic dermatitis, hidradenitis suppurativa and vitiligo. Cell Cycle.

[2] Egawa G., Kabashima K. (2011). Skin as a peripheral lymphoid organ: Revisiting the concept of skin-associated lymphoid tissues. J. Investig. Dermatol..

[3] Sawada Y., Nakamura M., Tokura Y. (2011). Generalized fixed drug eruption caused by pazufloxacin. Acta Derm.-Venereol..

[4] Tomura M., Honda T., Tanizaki H., Otsuka A., Egawa G., Tokura Y., Waldmann H., Hori S., Cyster J.G., Watanabe T. (2010). Activated regulatory T cells are the major T cell type emigrating from the skin during a cutaneous immune response in mice. J. Clin. Investig..

[5] Sawada Y., Gallo R.L. (2021). Role of Epigenetics in the Regulation of Immune Functions of the Skin. J. Investig. Dermatol..

[6] Kabashima K., Honda T., Ginhoux F., Egawa G. (2019). The immunological anatomy of the skin. Nat. Rev. Immunol..

[7] Sawada Y., Nakatsuji T., Dokoshi T., Kulkarni N.N., Liggins M.C., Sen G., Gallo R.L. (2021). Cutaneous innate immune tolerance is mediated by epigenetic control of MAP2K3 by HDAC8/9. Sci. Immunol..

[8] Alavi A., Anooshirvani N., Kim W.B., Coutts P., Sibbald R.G. (2015). Quality-of-life impairment in patients with hidradenitis suppurativa: A Canadian study. Am. J. Clin. Dermatol..

[9] Onderdijk A.J., van der Zee H.H., Esmann S., Lophaven S., Dufour D.N., Jemec G.B., Boer J. (2013). Depression in patients with hidradenitis suppurativa. J. Eur. Acad. Dermatol. Venereol..

[10] Prens E., Deckers I. (2015). Pathophysiology of hidradenitis suppurativa: An update. J. Am. Acad. Dermatol..

[11] Tchero H., Herlin C., Bekara F., Fluieraru S., Teot L. (2019). Hidradenitis Suppurativa: A Systematic Review and Meta-analysis of Therapeutic Interventions. Indian J. Dermatol. Venereol. Leprol..

[12] Garg A., Naik H.B., Kirby J.S. (2023). A Practical Guide for Primary Care Providers on Timely Diagnosis and Comprehensive Care Strategies for Hidradenitis Suppurativa. Am. J. Med..

[13] Itamura M., Sawada Y. (2022). Involvement of Atopic Dermatitis in the Development of Systemic Inflammatory Diseases. Int. J. Mol. Sci..

[14] Jung H.J., Lee D.H., Park M.Y., Ahn J. (2021). Cardiovascular comorbidities of atopic dermatitis: Using National Health Insurance data in Korea. Allergy Asthma Clin. Immunol..

[15] Ahlehoff O., Gislason G.H., Jørgensen C.H., Lindhardsen J., Charlot M., Olesen J.B., Abildstrøm S.Z., Skov L., Torp-Pedersen C., Hansen P.R. (2012). Psoriasis and risk of atrial fibrillation and ischaemic stroke: A Danish Nationwide Cohort Study. Eur. Heart J..

[16] Emelianov V.U., Bechara F.G., Gläser R., Langan E.A., Taungjaruwinai W.M., Schröder J.M., Meyer K.C., Paus R. (2012). Immunohistological pointers to a possible role for excessive cathelicidin (LL-37) expression by apocrine sweat glands in the pathogenesis of hidradenitis suppurativa/acne inversa. Br. J. Dermatol..

[17] Yu C.C., Cook M.G. (1990). Hidradenitis suppurativa: A disease of follicular epithelium, rather than apocrine glands. Br. J. Dermatol..

[18] Von Laffert M., Stadie V., Wohlrab J., Marsch W.C. (2011). Hidradenitis suppurativa/acne inversa: Bilocated epithelial hyperplasia with very different sequelae. Br. J. Dermatol..

[19] Schlapbach C., Hänni T., Yawalkar N., Hunger R.E. (2011). Expression of the IL-23/Th17 pathway in lesions of hidradenitis suppurativa. J. Am. Acad. Dermatol..

[20] Van der Zee H.H., Laman J.D., de Ruiter L., Dik W.A., Prens E.P. (2012). Adalimumab (antitumour necrosis factor-α) treatment of hidradenitis suppurativa ameliorates skin inflammation: An in situ and ex vivo study. Br. J. Dermatol..

[21] Byrd A.S., Carmona-Rivera C., O’Neil L.J., Carlucci P.M., Cisar C., Rosenberg A.Z., Kerns M.L., Caffrey J.A., Milner S.M., Sacks J.M. (2019). Neutrophil extracellular traps, B cells, and type I interferons contribute to immune dysregulation in hidradenitis suppurativa. Sci. Transl. Med..

[22] Thomi R., Cazzaniga S., Seyed Jafari S.M., Schlapbach C., Hunger R.E. (2018). Association of Hidradenitis Suppurativa with T Helper 1/T Helper 17 Phenotypes: A Semantic Map Analysis. JAMA Dermatol..

[23] Di Caprio R., Balato A., Caiazzo G., Lembo S., Raimondo A., Fabbrocini G., Monfrecola G. (2017). IL-36 cytokines are increased in acne and hidradenitis suppurativa. Arch. Dermatol. Res..

[24] Schlapbach C., Yawalkar N., Hunger R.E. (2009). Human beta-defensin-2 and psoriasin are overexpressed in lesions of acne inversa. J. Am. Acad. Dermatol..

[25] Yang M.S., Park E.J., Sohn S., Kwon H.J., Shin W.H., Pyo H.K., Jin B., Choi K.S., Jou I., Joe E.H. (2002). Interleukin-13 and -4 induce death of activated microglia. Glia.

[26] Steinman L. (2008). A rush to judgment on Th17. J. Exp. Med..

[27] Das R., Chen X., Komorowski R., Hessner M.J., Drobyski W.R. (2009). Interleukin-23 secretion by donor antigen-presenting cells is critical for organ-specific pathology in graft-versus-host disease. Blood.

[28] Grant A., Gonzalez T., Montgomery M.O., Cardenas V., Kerdel F.A. (2010). Infliximab therapy for patients with moderate to severe hidradenitis suppurativa: A randomized, double-blind, placebo-controlled crossover trial. J. Am. Acad. Dermatol..

[29] Kridin K., Valido K., Cohen J.M., Cohen A.D. (2022). Hidradenitis suppurativa and the risk of myocardial infarction, cerebrovascular accident, and peripheral vascular disease: A population-based study. Arch. Dermatol. Res..

[30] Egeberg A., Gislason G.H., Hansen P.R. (2016). Risk of Major Adverse Cardiovascular Events and All-Cause Mortality in Patients with Hidradenitis Suppurativa. JAMA Dermatol..

[31] Reddy S., Strunk A., Jemec G.B.E., Garg A. (2020). Incidence of Myocardial Infarction and Cerebrovascular Accident in Patients with Hidradenitis Suppurativa. JAMA Dermatol..

[32] Hung C.T., Chiang C.P., Chung C.H., Tsao C.H., Chien W.C., Wang W.M. (2019). Increased risk of cardiovascular comorbidities in hidradenitis suppurativa: A nationwide, population-based, cohort study in Taiwan. J. Dermatol..

[33] González-López M.A., Hernández J.L., Lacalle M., Mata C., López-Escobar M., López-Mejías R., Portilla V., Fuentevilla P., Corrales A., González-Vela M.C. (2016). Increased prevalence of subclinical atherosclerosis in patients with hidradenitis suppurativa (HS). J. Am. Acad. Dermatol..

[34] Pascual J.C., González I., Corona D., Hispán P., Ramos J.M., Sánchez-Paya J., Jemec G.B. (2017). Assessment of subclinical atherosclerosis in hidradenitis suppurativa. J. Eur. Acad. Dermatol. Venereol..

[35] Paramel Varghese G., Folkersen L., Strawbridge R.J., Halvorsen B., Yndestad A., Ranheim T., Krohg-Sørensen K., Skjelland M., Espevik T., Aukrust P. (2016). NLRP3 Inflammasome Expression and Activation in Human Atherosclerosis. J. Am. Heart Assoc..

[36] Ridker P.M., Howard C.P., Walter V., Everett B., Libby P., Hensen J., Thuren T. (2012). Effects of interleukin-1β inhibition with canakinumab on hemoglobin A1c, lipids, C-reactive protein, interleukin-6, and fibrinogen: A phase IIb randomized, placebo-controlled trial. Circulation.

[37] Ridker P.M., Everett B.M., Thuren T., MacFadyen J.G., Chang W.H., Ballantyne C., Fonseca F., Nicolau J., Koenig W., Anker S.D. (2017). Antiinflammatory Therapy with Canakinumab for Atherosclerotic Disease. N. Engl. J. Med..

[38] Eid R.E., Rao D.A., Zhou J., Lo S.F., Ranjbaran H., Gallo A., Sokol S.I., Pfau S., Pober J.S., Tellides G. (2009). Interleukin-17 and interferon-gamma are produced concomitantly by human coronary artery-infiltrating T cells and act synergistically on vascular smooth muscle cells. Circulation.

[39] Karbach S., Croxford A.L., Oelze M., Schüler R., Minwegen D., Wegner J., Koukes L., Yogev N., Nikolaev A., Reißig S. (2014). Interleukin 17 drives vascular inflammation, endothelial dysfunction, and arterial hypertension in psoriasis-like skin disease. Arterioscler. Thromb. Vasc. Biol..

[40] Schonmann Y., Mansfield K.E., Mulick A., Roberts A., Smeeth L., Langan S.M., Nitsch D. (2021). Inflammatory skin diseases and the risk of chronic kidney disease: Population-based case-control and cohort analyses. Br. J. Dermatol..

[41] Chung B.H., Kim K.W., Sun I.O., Choi S.R., Park H.S., Jeon E.J., Kim B.M., Choi B.S., Park C.W., Kim Y.S. (2012). Increased interleukin-17 producing effector memory T cells in the end-stage renal disease patients. Immunol. Lett..

[42] Lavoz C., Matus Y.S., Orejudo M., Carpio J.D., Droguett A., Egido J., Mezzano S., Ruiz-Ortega M. (2019). Interleukin-17A blockade reduces albuminuria and kidney injury in an accelerated model of diabetic nephropathy. Kidney Int..

[43] Odobasic D., Gan P.Y., Summers S.A., Semple T.J., Muljadi R.C., Iwakura Y., Kitching A.R., Holdsworth S.R. (2011). Interleukin-17A promotes early but attenuates established disease in crescentic glomerulonephritis in mice. Am. J. Pathol..

[44] Gisondi P., Targher G., Zoppini G., Girolomoni G. (2009). Non-alcoholic fatty liver disease in patients with chronic plaque psoriasis. J. Hepatol..

[45] Damiani G., Leone S., Fajgenbaum K., Bragazzi N.L., Pacifico A., Conic R.R., Pigatto P.D., Maiorana C., Poli P., Berti E. (2019). Nonalcoholic fatty liver disease prevalence in an Italian cohort of patients with hidradenitis suppurativa: A multi-center retrospective analysis. World J. Hepatol..

[46] Durán-Vian C., Arias-Loste M.T., Hernández J.L., Fernández V., González M., Iruzubieta P., Rasines L., González-Vela C., Vaqué J.P., Blanco R. (2019). High prevalence of non-alcoholic fatty liver disease among hidradenitis suppurativa patients independent of classic metabolic risk factors. J. Eur. Acad. Dermatol. Venereol..

[47] González-Villanueva I., DeGracia C., Planells M., Poveda I., Álvarez P., Schneller-Pavalescu L., Betlloch I., Jemec G.B.E., Ramos J.M., Pascual J.C. (2020). Hidradenitis Suppurativa is Associated with Non-alcoholic Fatty Liver Disease: A Cross-sectional Study. Acta Derm.-Venereol..

[48] He B., Wu L., Xie W., Shao Y., Jiang J., Zhao Z., Yan M., Chen Z., Cui D. (2017). The imbalance of Th17/Treg cells is involved in the progression of nonalcoholic fatty liver disease in mice. BMC Immunol..

[49] Li F., Hao X., Chen Y., Bai L., Gao X., Lian Z., Wei H., Sun R., Tian Z. (2017). The microbiota maintain homeostasis of liver-resident γδT-17 cells in a lipid antigen/CD1d-dependent manner. Nat. Commun..

[50] Tang Y., Bian Z., Zhao L., Liu Y., Liang S., Wang Q., Han X., Peng Y., Chen X., Shen L. (2011). Interleukin-17 exacerbates hepatic steatosis and inflammation in non-alcoholic fatty liver disease. Clin. Exp. Immunol..

[51] Gomes A.L., Teijeiro A., Burén S., Tummala K.S., Yilmaz M., Waisman A., Theurillat J.P., Perna C., Djouder N. (2016). Metabolic Inflammation-Associated IL-17A Causes Non-alcoholic Steatohepatitis and Hepatocellular Carcinoma. Cancer Cell.

[52] Rolla S., Alchera E., Imarisio C., Bardina V., Valente G., Cappello P., Mombello C., Follenzi A., Novelli F., Carini R. (2016). The balance between IL-17 and IL-22 produced by liver-infiltrating T-helper cells critically controls NASH development in mice. Clin. Sci..

[53] Choi J.H., Han J., Theodoropoulos P.C., Zhong X., Wang J., Medler D., Ludwig S., Zhan X., Li X., Tang M. (2020). Essential requirement for nicastrin in marginal zone and B-1 B cell development. Proc. Natl. Acad. Sci. USA.

[54] Pan Y., Lin M.H., Tian X., Cheng H.T., Gridley T., Shen J., Kopan R. (2004). Gamma-secretase functions through Notch signaling to maintain skin appendages but is not required for their patterning or initial morphogenesis. Dev. Cell.

[55] Pink A.E., Simpson M.A., Brice G.W., Smith C.H., Desai N., Mortimer P.S., Barker J.N., Trembath R.C. (2011). PSENEN and NCSTN mutations in familial hidradenitis suppurativa (Acne Inversa). J. Investig. Dermatol..

[56] Esme P., Esme M., Caliskan E. (2020). Increased prevalence of family history of Alzheimer's disease in hidradenitis suppurativa: Cross-sectional analysis of 192 HS patients. Dermatol. Ther..

[57] Garg A., Strunk A. (2017). Risk of Alzheimer’s disease is not increased among patients with hidradenitis suppurativa: A retrospective population-based cohort analysis. J. Am. Acad. Dermatol..

[58] Oberstein T.J., Taha L., Spitzer P., Hellstern J., Herrmann M., Kornhuber J., Maler J.M. (2018). Imbalance of Circulating T(h)17 and Regulatory T Cells in Alzheimer’s Disease: A Case Control Study. Front. Immunol..

[59] Zhang J., Ke K.F., Liu Z., Qiu Y.H., Peng Y.P. (2013). Th17 cell-mediated neuroinflammation is involved in neurodegeneration of aβ1-42-induced Alzheimer’s disease model rats. PLoS ONE.

[60] Cristiano C., Volpicelli F., Lippiello P., Buono B., Raucci F., Piccolo M., Iqbal A.J., Irace C., Miniaci M.C., Perrone Capano C. (2019). Neutralization of IL-17 rescues amyloid-β-induced neuroinflammation and memory impairment. Br. J. Pharmacol..

[61] Xu J.J., Guo S., Xue R., Xiao L., Kou J.N., Liu Y.Q., Han J.Y., Fu J.J., Wei N. (2021). Adalimumab ameliorates memory impairments and neuroinflammation in chronic cerebral hypoperfusion rats. Aging.

[62] Tashiro T., Sawada Y. (2022). Psoriasis and Systemic Inflammatory Disorders. Int. J. Mol. Sci..

[63] Miller I.M., Rytgaard H., Mogensen U.B., Miller E., Ring H.C., Ellervik C., Jemec G.B. (2016). Body composition and basal metabolic rate in Hidradenitis Suppurativa: A Danish population-based and hospital-based cross-sectional study. J. Eur. Acad. Dermatol. Venereol..

[64] Fauconier M., Reguiai Z., Barbe C., Colosio A., Eschard J.P., Salmon J.H., Direz G. (2018). Association between hidradenitis suppurativa and spondyloarthritis. Jt. Bone Spine.

[65] Navarro I., González-López M.A., Sierra I., Olmos J.M., Blanco R., Hernández J.L. (2022). Bone Metabolism in Patients with Hidradenitis Suppurativa: A Case-control Study. Acta Derm.-Venereol..

[66] Kastelan D., Kastelan M., Massari L.P., Korsic M. (2006). Possible association of psoriasis and reduced bone mineral density due to increased TNF-alpha and IL-6 concentrations. Med. Hypotheses.

[67] Kotake S., Udagawa N., Takahashi N., Matsuzaki K., Itoh K., Ishiyama S., Saito S., Inoue K., Kamatani N., Gillespie M.T. (1999). IL-17 in synovial fluids from patients with rheumatoid arthritis is a potent stimulator of osteoclastogenesis. J. Clin. Investig..

[68] Shukla P., Mansoori M.N., Singh D. (2018). Efficacy of anti-IL-23 monotherapy versus combination therapy with anti-IL-17 in estrogen deficiency induced bone loss conditions. Bone.

[69] Reddy S., Strunk A., Garg A. (2019). Comparative Overall Comorbidity Burden Among Patients with Hidradenitis Suppurativa. JAMA Dermatol..

[70] Prens L.M., Bouwman K., Troelstra L.D., Prens E.P., Alizadeh B.Z., Horváth B. (2022). New insights in hidradenitis suppurativa from a population-based Dutch cohort: Prevalence, smoking behaviour, socioeconomic status and comorbidities. Br. J. Dermatol..

[71] Duan M.C., Tang H.J., Zhong X.N., Huang Y. (2013). Persistence of Th17/Tc17 cell expression upon smoking cessation in mice with cigarette smoke-induced emphysema. Clin. Dev. Immunol..

[72] Chen Y., Kumar R.K., Thomas P.S., Herbert C. (2019). Th1/17-Biased Inflammatory Environment Associated with COPD Alters the Response of Airway Epithelial Cells to Viral and Bacterial Stimuli. Mediat. Inflamm..

[73] Saygın D., Syed A.U., Lowder C.Y., Srivastava S., Maya J.J., Hajj-Ali R.A. (2018). Characteristics of inflammatory eye disease associated with hidradenitis suppurativa. Eur. J. Rheumatol..

[74] Lee D.J., Desai S., Laurent E., Kopplin L.J. (2021). Characterization and Management of Inflammatory Eye Disease in Patients with Hidradenitis Suppurativa. Ocul. Immunol. Inflamm..

[75] Amadi-Obi A., Yu C.R., Liu X., Mahdi R.M., Clarke G.L., Nussenblatt R.B., Gery I., Lee Y.S., Egwuagu C.E. (2007). TH17 cells contribute to uveitis and scleritis and are expanded by IL-2 and inhibited by IL-27/STAT1. Nat. Med..

[76] Yoshimura T., Sonoda K.H., Ohguro N., Ohsugi Y., Ishibashi T., Cua D.J., Kobayashi T., Yoshida H., Yoshimura A. (2009). Involvement of Th17 cells and the effect of anti-IL-6 therapy in autoimmune uveitis. Rheumatology.

[77] Tang J., Zhou R., Luger D., Zhu W., Silver P.B., Grajewski R.S., Su S.B., Chan C.C., Adorini L., Caspi R.R. (2009). Calcitriol suppresses antiretinal autoimmunity through inhibitory effects on the Th17 effector response. J. Immunol..

[78] Egeberg A., Jemec G.B.E., Kimball A.B., Bachelez H., Gislason G.H., Thyssen J.P., Mallbris L. (2017). Prevalence and Risk of Inflammatory Bowel Disease in Patients with Hidradenitis Suppurativa. J. Investig. Dermatol..

[79] Leppkes M., Becker C., Ivanov I.I., Hirth S., Wirtz S., Neufert C., Pouly S., Murphy A.J., Valenzuela D.M., Yancopoulos G.D. (2009). RORgamma-expressing Th17 cells induce murine chronic intestinal inflammation via redundant effects of IL-17A and IL-17F. Gastroenterology.

[80] Fina D., Sarra M., Fantini M.C., Rizzo A., Caruso R., Caprioli F., Stolfi C., Cardolini I., Dottori M., Boirivant M. (2008). Regulation of gut inflammation and th17 cell response by interleukin-21. Gastroenterology.

[81] Schmechel S., Konrad A., Diegelmann J., Glas J., Wetzke M., Paschos E., Lohse P., Göke B., Brand S. (2008). Linking genetic susceptibility to Crohn’s disease with Th17 cell function: IL-22 serum levels are increased in Crohn's disease and correlate with disease activity and IL23R genotype status. Inflamm. Bowel Dis..

[82] Sherman S., Tzur Bitan D., Kridin K., Pavlovsky L., Hodak E., Cohen A.D. (2021). Hidradenitis suppurativa is associated with hypothyroidism and hyperthyroidism: A large-scale population-based study. Int. J. Dermatol..

[83] Nanba T., Watanabe M., Inoue N., Iwatani Y. (2009). Increases of the Th1/Th2 cell ratio in severe Hashimoto’s disease and in the proportion of Th17 cells in intractable Graves’ disease. Thyroid.

[84] Konca Degertekin C., Aktas Yilmaz B., Balos Toruner F., Kalkanci A., Turhan Iyidir O., Fidan I., Yesilyurt E., Cakır N., Kustimur S., Arslan M. (2016). Circulating Th17 cytokine levels are altered in Hashimoto’s thyroiditis. Cytokine.

[85] Olivieri A.N., Iafusco D., Mellos A., Zanfardino A., Mauro A., Granato C., Gicchino M.F., Prisco F., Perrone L. (2013). Refractory rheumatoid factor positive polyarthritis in a female adolescent already suffering from type 1 diabetes mellitus and Hashimoto’s thyroiditis successfully treated with etanercept. Ital. J. Pediatr..

[86] Lee J.H., Kwon H.S., Jung H.M., Kim G.M., Bae J.M. (2018). Prevalence and comorbidities associated with hidradenitis suppurativa in Korea: A nationwide population-based study. J. Eur. Acad. Dermatol. Venereol..

[87] Horissian M., Maczuga S., Kirby J.S., Nelson A.M. (2019). Increased risk of alopecia areata for people with hidradenitis suppurativa in a cross-sectional study. J. Am. Acad. Dermatol..

[88] Ito T., Hashizume H., Shimauchi T., Funakoshi A., Ito N., Fukamizu H., Takigawa M., Tokura Y. (2013). CXCL10 produced from hair follicles induces Th1 and Tc1 cell infiltration in the acute phase of alopecia areata followed by sustained Tc1 accumulation in the chronic phase. J. Dermatol. Sci..

[89] Suárez-Fariñas M., Ungar B., Noda S., Shroff A., Mansouri Y., Fuentes-Duculan J., Czernik A., Zheng X., Estrada Y.D., Xu H. (2015). Alopecia areata profiling shows TH1, TH2 and IL-23 cytokine activation without parallel TH17/TH22 skewing. J. Allergy Clin. Immunol..

[90] Tanemura A., Oiso N., Nakano M., Itoi S., Kawada A., Katayama I. (2013). Alopecia areata: Infiltration of Th17 cells in the dermis, particularly around hair follicles. Dermatology.

[91] Gau S.Y., Preclaro I.A.C., Wei J.C., Lee C.Y., Kuan Y.H., Hsiao Y.P., Juang S.E., Ma K.S. (2022). Risk of psoriasis in people with hidradenitis suppurativa: A systematic review and meta-analysis. Front. Immunol..

[92] Thorlacius L., Cohen A.D., Gislason G.H., Jemec G.B.E., Egeberg A. (2018). Increased Suicide Risk in Patients with Hidradenitis Suppurativa. J. Investig. Dermatol..

[93] Wright S., Strunk A., Garg A. (2020). New-onset depression among children, adolescents, and adults with hidradenitis suppurativa. J. Am. Acad. Dermatol..

[94] Westfall S., Caracci F., Zhao D., Wu Q.L., Frolinger T., Simon J., Pasinetti G.M. (2021). Microbiota metabolites modulate the T helper 17 to regulatory T cell (Th17/Treg) imbalance promoting resilience to stress-induced anxiety- and depressive-like behaviors. Brain Behav. Immun..

[95] Kim J., Suh Y.H., Chang K.A. (2021). Interleukin-17 induced by cumulative mild stress promoted depression-like behaviors in young adult mice. Mol. Brain.

[96] Pavón L., Sandoval-López G., Eugenia Hernández M., Loría F., Estrada I., Pérez M., Moreno J., Avila U., Leff P., Antón B. (2006). Th2 cytokine response in Major Depressive Disorder patients before treatment. J. Neuroimmunol..

[97] Miniotti M., Lazzarin G., Ortoncelli M., Mastorino L., Ribero S., Leombruni P. (2022). Impact on health-related quality of life and symptoms of anxiety and depression after 32 weeks of Dupilumab treatment for moderate-to-severe atopic dermatitis. Dermatol. Ther..

[98] Lönndahl L., Lundqvist M., Bradley M., Johansson E.K. (2022). Dupilumab Significantly Reduces Symptoms of Prurigo Nodularis and Depression: A Case Series. Acta Derm.-Venereol..

[99] Tzellos T., Zouboulis C.C., Gulliver W., Cohen A.D., Wolkenstein P., Jemec G.B. (2015). Cardiovascular disease risk factors in patients with hidradenitis suppurativa: A systematic review and meta-analysis of observational studies. Br. J. Dermatol..

[100] Vasseur P., Serres L., Jégou J.F., Pohin M., Delwail A., Petit-Paris I., Levillain P., Favot L., Samson M., Yssel H. (2016). High-Fat Diet-Induced IL-17A Exacerbates Psoriasiform Dermatitis in a Mouse Model of Steatohepatitis. Am. J. Pathol..

[101] Savetsky I.L., Albano N.J., Cuzzone D.A., Gardenier J.C., Torrisi J.S., García Nores G.D., Nitti M.D., Hespe G.E., Nelson T.S., Kataru R.P. (2015). Lymphatic Function Regulates Contact Hypersensitivity Dermatitis in Obesity. J. Investig. Dermatol..

[102] Nakamizo S., Honda T., Adachi A., Nagatake T., Kunisawa J., Kitoh A., Otsuka A., Dainichi T., Nomura T., Ginhoux F. (2017). High fat diet exacerbates murine psoriatic dermatitis by increasing the number of IL-17-producing γδ T cells. Sci. Rep..

[103] Lapins J., Ye W., Nyrén O., Emtestam L. (2001). Incidence of cancer among patients with hidradenitis suppurativa. Arch. Dermatol..

[104] Jung J.M., Lee K.H., Kim Y.J., Chang S.E., Lee M.W., Choi J.H., Won C.H., Lee W.J. (2020). Assessment of Overall and Specific Cancer Risks in Patients with Hidradenitis Suppurativa. JAMA Dermatol..

[105] Morimura S., Sugaya M., Oka T., Suga H., Miyagaki T., Tsunemi Y., Asano Y., Sato S. (2021). Increased Regulatory T Cells and Decreased Myeloid-Derived Suppressor Cells Induced by High CCL17 Levels May Account for Normal Incidence of Cancers among Patients with Atopic Dermatitis. Int. J. Mol. Sci..

[106] Villani A.P., Pavel A.B., Wu J., Fernandes M., Maari C., Saint-Cyr Proulx E., Jack C., Glickman J., Choi S., He H. (2021). Vascular inflammation in moderate-to-severe atopic dermatitis is associated with enhanced Th2 response. Allergy.

[107] Gelfand J.M., Shin D.B., Duffin K.C., Armstrong A.W., Blauvelt A., Tyring S.K., Menter A., Gottlieb S., Lockshin B.N., Simpson E.L. (2020). A Randomized Placebo-Controlled Trial of Secukinumab on Aortic Vascular Inflammation in Moderate-to-Severe Plaque Psoriasis (VIP-S). J. Investig. Dermatol..

